# Patients as partners in health research: A scoping review

**DOI:** 10.1111/hex.13272

**Published:** 2021-06-21

**Authors:** Tamara L. McCarron, Fiona Clement, Jananee Rasiah, Chelsea Moran, Karen Moffat, Andrea Gonzalez, Tracy Wasylak, Maria Santana

**Affiliations:** ^1^ The Department Community Health Sciences Calgary AB Canada; ^2^ O’Brien Institute for Public Health Calgary AB Canada; ^3^ Faculty of Nursing 3‐141 Edmonton Clinic Health Academy (ECHA) University of Alberta Edmonton AB Canada; ^4^ Patient Partner Calgary AB Canada; ^5^ The Department Psychology University of Calgary Calgary AB Canada; ^6^ Alberta Health Services Calgary AB Canada; ^7^ Faculty of Nursing University of Calgary Calgary AB Canada

**Keywords:** co‐production, patient and public involvement, patient engagement, scoping review

## Abstract

**Background:**

The role of patient involvement in health research has evolved over the past decade. Despite efforts to engage patients as partners, the role is not well understood. We undertook this review to understand the engagement practices of patients who assume roles as partners in health research.

**Methods:**

Using a recognized methodological approach, two academic databases (MEDLINE and EMBASE) and grey literature sources were searched. Findings were organized into one of the three higher levels of engagement, described by the Patient and Researcher Engagement framework developed by Manafo. We examined and quantified the supportive strategies used during involvement, used thematic analysis as described by Braun and Clarke and themed the purpose of engagement, and categorized the reported outcomes according to the CIHR Engagement Framework.

**Results:**

Out of 6621 records, 119 sources were included in the review. Thematic analysis of the purpose of engagement revealed five themes: documenting and advancing PPI, relevance of research, co‐building, capacity building and impact on research. Improved research design was the most common reported outcome and the most common role for patient partners was as members of the research team, and the most commonly used strategy to support involvement was by meetings.

**Conclusion:**

The evidence collected during this review advanced our understanding of the engagement of patients as research partners. As patient involvement becomes more mainstream, this knowledge will aid researchers and policy‐makers in the development of approaches and tools to support engagement.

**Patient/User Involvement:**

Patients led and conducted the grey literature search, including the synthesis and interpretation of the findings.

## 1 BACKGROUND

The involvement of patients in health research is an emerging phenomenon, with critical barriers still present for researchers seeking to involve patients as partners.^1^ Globally, patient and public involvement (PPI) organizations such as INVOLVE in the United Kingdom,^2^ Patient‐Centered Outcomes Research Institute (PCORI) in the United States^3^ and the Canadian Institutes for Health Research (CIHR) Strategy for Patient‐Oriented Research (SPOR) in Canada^4^ have been established. These organizations aim to support the involvement of patients, their caregivers and families in the research process by promoting and providing infrastructure to support PPI in health research.

As such, the role of patients and how they engage in research has evolved over the past decade. CIHR defines this new role as “patient partner” and describes when patients contribute to the research process and research‐related activities, different from the traditional, more passive role, as research participant, where patients receive treatments or are under observation.^5^ Patient partners are more involved in conducting research activities, at all stages of the research process, including supporting grant applications, assisting with participant recruitment and performing research dissemination activities.^6^ In Canada, the SPOR patient engagement framework encompasses four principles to guide the involvement of patients as partners in research. These include the following: (a) ensuring inclusivity, so that perspectives of patients who may otherwise be excluded, marginalized, hard to reach or are members of minority groups are invited; (b) providing support through flexible environments with training opportunities that will meet patients’ diverse needs; (c) promoting mutual respect by valuing patients’, researchers’ and clinicians’ perspectives through regular discussions; and (d) co‐building research projects by identifying priorities and gaps and working to implement solutions together.^7^ Equally important in supporting patient involvement, this same framework emphasizes six outcomes for patient engagement including (a) multi‐way capacity building, which ensures that the support needed for patients and researchers to be able to work together are in place; (b) inclusive mechanisms and processes, which supports an environment where patient engagement can occur at all levels; (c) multi‐way communication and collaboration, which describes a safe environment of mutual respect; (d) patient‐informed and directed research, which discuss supportive research approaches that engage a diversity of patients; (e) experiential knowledge is valued as evidence, recognizing the importance of lived experience and the ability of this knowledge to be mobilized and translated; and (f) a shared sense of purpose, where participants, both patient and researcher, work together towards a common goal.^7^

Bird et al^1^ conducted a scoping review exploring the impact of patient partners on research outcomes, acknowledging the results of this review primarily involved adults with chronic, long‐term conditions, a number of critical barriers and facilitators for researcher seeking to undertake patient partnerships were identified. Hoekstra et al synthesized the research partnership literature, and recommended a four step guidance to support partnerships processes including (a) building and maintaining relationship; (b) determining the appropriate level of engagement for each phase of the research process; (c) selecting or adapting strategies to the desired level of engagement; and (d) communicate and report the principles, strategies, outcomes and impacts of the research partnerships.^8^

Broadly, there is an understanding of researcher motivations to engage patients in the research process, such as improving the relevance of research,^9^ and an understanding of the motivations of patients who are involved as partners, such as improving health care.^10^ Systematic reviews on the involvement and engagement of patients in research have not differentiated between the evolving role of patient as partner in research and patient as research participant.^9^, ^11^ Currently, there is little peer‐reviewed published literature that differentiates the role of patient as partner in research from the role of patients as research participant.^12^ Although PPI has been shown to develop on‐going, productive and mutually advantageous relationships between researchers and patients,^13^, ^14^ the role of patient as research partner is not well understood. While the evidence provided by Manafo et al^15^ describes the characteristics of patient as partners, we conducted this review to understand the engagement of patients who assume roles as partners in health research projects. Since “patient as partner” is defined by individuals who assume roles with greater involvement, increased sharing of power and increased responsibility on research teams,^16^ we focused the review at the higher levels of the engagement spectrum (involve, collaborate and lead/support) as defined by Manafo et al^15^ The specific objectives of this review were to (a) report the purpose of engagement; (b) report the outcomes of engagement; (c) to capture the patient role as described by the study author; and (d) to explore the engagement strategies to support patient involvement.

## 2 METHODS

A scoping review was conducted because this body of literature has not yet been comprehensively reviewed, it is heterogeneous in nature and therefore not amenable to a more precise systematic review.

### 2.1 Design

We followed the methodological framework proposed by Arksey and O’Malley^17^ and further enhanced by Levac et al^18^ This six‐stage methodological framework includes (a) identifying the research question, (b) identifying relevant studies, (c) study selection, (d) charting the data, (e) collating, summarizing and reporting the results and (f) stakeholder consultation. A scoping review methodology was chosen to identify the research in how patient partners are involved as patient partners in health research.

### 2.2 Recruitment of patient partners

Posters were distributed among the first authors’ personal networks, and other organizations including the Alberta SPOR Support Unit^19^ and Albertans for Health Research.^20^ The project description, proposed role and anticipated time commitment were included. Five individuals responded to the opportunity. All five individuals were interviewed and later selected. These individuals represented a diverse group in terms of diversity, age and sex and had varying experience working on research projects (from no experience to some experience).

Ethic approval was not needed as these patients were members of the research team rather than participants of the study. The details about the engagement process are described somewhere else.^21^

### 2.3 Stage 1: Identifying the research question

Our research team, including our patient partners and key stakeholders such as the Strategic Clinical Networks™ at Alberta Health Services, developed the research question guiding this review: “How are patients engaged as partners in health research?” We used a modified SPICE (setting, population/perspective, intervention, comparison and evaluation) methodology to develop our research question.^22^

### 2.4 Stage 2: Identifying relevant studies

#### 2.4.1 Protocol and registration

The final review protocol was developed a priori and posted on the Open Science Framework (https://osf.io/h2p8s/). This review was completed in accordance with the scoping review reporting guidelines (PRISMA‐ScR).^23^

#### 2.4.2 Eligibility criteria

To be included in the review, studies were considered if they: described patients assuming roles in health research at one of the six levels as defined by the spectrum of Patient and Researcher Engagement described by Manafo et al^15^; were written in English; and were published between 1 January 2010 and 14 January 2020. This time period was chosen to capture the evolving role of patients who began assuming responsibilities as partners in research. Studies written in English were considered given the limitations of the research team. Studies were excluded if they were an opinion, editorial, did not involve or engage patients, did not describe the engagement of patients or were examples of community rather than patient engagement.

#### 2.4.3 Information sources

Three sources of data informed our scoping review: an academic database search; grey literature search; and hand‐searching of reference list of identified studies. Our academic search strategy was developed in consultation with a health sciences research librarian and was applied to the MEDLINE and EMBASE databases. Search terms were determined with input from the research team, research collaborators and our team of patient partners and included a broad combination of title and keyword search terms such as “patient”, “consumer”, “carer”, “family” and “community” and mesh headings such as “Patient Participation” and “Health Services Research” (Appendix S1). A team of patient partners led and conducted the grey literature component of this review. Patient partners identified studies, reports and conference abstracts of relevance to this review. To ensure that all relevant information was captured, we modified the CADTH Grey Matters^24^ tool for searching grey literature to include an exhaustive list of organizations with a mandate in the area of patient engagement such as INVOLVE in the United Kingdom and SPOR in Canada (Appendix S2). Two training sessions on developing and conducting a grey literature coupled with weekly discussions and support meetings were held with the patient partners. A custom Google search was conducted using a comprehensive list of search terms such as “patient participation”, “public involvement” and “community integration” (Appendix S3). Finally, we hand‐searched reference lists of identified reviews to identify additional studies of relevance.

### 2.5 Stage 3: Study selection

#### 2.5.1 Selection of sources of evidence

The review process consisted of two levels of screening: (a) a title and abstract review and (b) full‐text screening to assign studies to a level of engagement. For the title and abstract review, the team calibrated on the first 100, until a high level of agreement between reviewers was reached. Four reviewers, in two teams of two, independently screened all title and abstracts. When determining eligibility at full‐text screening, only studies that described the higher levels of Patient and Researcher Engagement Spectrum were retained such as at the involve level, which involved patients as members of an advisory group, the collaborate level, which described partnering as a team member; or the lead/support level which described patients leading research activities.^15^ Thus, studies underwent an additional screening process where two independent reviewers assigned each study to a level of engagement according to the authors’ report of the engagement activities.^15^ Articles reporting more than one level of engagement method were assigned to the higher level. The level of engagement of included articles was confirmed during the data extraction process, and any disagreements were resolved by a third reviewer.

### 2.6 Stage 4: Data collection

#### 2.6.1 Data charting

The same data collection instruments for both the grey and academic sources were developed a priori by the research team to confirm study relevance and to extract study characteristics. Study characteristics were recorded in tabular form (using frequencies/percentages) with the following article characteristics: (a) country of origin; (b) author; (c) study design; (d) purpose of engagement as reported by the study author; (e) study population as reported in the manuscript; (f) type of engagement activities listed in the study; (g) purpose/goal of engagement activities as listed in the study; and (h) any reported direct/indirect study outcome(s) as a result of the engagement activity. Patient partner characteristics were also collected including (a) age; (b) sex; (c) gender; (d) actor (patient/parent/family member/caregiver/community member); (e) ethnicity; (f) level of education; (g) compensation received; and (h) engagement training received (Appendix S4).

Data extraction was completed by three reviewers, one of which was a patient partner who extracted data from all included sources from the grey literature search. All data were verified by a fourth reviewer. The data were compiled in Microsoft Excel. Weekly team meetings were conducted to discuss articles and maintain reliability and quality during the data extraction process.

### 2.7 Stage 5: Data summary and synthesis

We used the six stages of thematic data analysis as described by Braun and Clarke, to ***report the purpose of engagement***: (a) familiarization; (b) initial coding; (c) identifying themes; (d) reviewing themes; (e) defining themes; and (f) reporting.^25^ To facilitate the theming process, the reported purpose of engagement of each of the included studies was imported into NVIVO 12. Research team members reviewed the data individually to generate initial codes. Theming was then completed as larger group and later verified by our patient partner. Using the Canadian Institutes for Health Research (CIHR) Patient Oriented Research (POR) strategy as a lens, the **reported outcomes** from each of the studies were categorized according to the six components of successful patient engagement including (a) *inclusive mechanisms and processes*, which is described by an environment where patient engagement can occur at all levels; (b) *multi‐way capacity building*, described as the support needed for patients and researchers to be able to work together; (c) *multi‐way communication and collaboration*, which is described when a safe environment of mutual respect is present; (d) *experiential knowledge of patients valued as evidence*, where the importance of lived experience is mobilized and translated; (e) *patient‐informed and directed research*, described by research approaches that engaged a diversity of patients; and (f) *a shared sense of purpose*, which is described by participants working together towards a common goal.^7^ Next, ***the patient role***, as identified by the study author, was captured. Lastly, **the strategies used to support patient partners** during their engagement, such as focus groups and face‐to‐face meetings, were determined using the word frequency query in NVIVO 12.

### 2.8 Stage 6: Consultation

According to the Scoping Review enhancements suggested by Levac,^18^ two consultation meetings were held with the patient partners, members of the Alberta SPOR Patient Engagement Platform^26^ such as the Patient Engagement Research Lead, and the broader research team including students and academics. The first meeting occurred at the beginning of the project where attendees reviewed and provided comments to the study question and the overall search strategy including the search terms. The second consultation meeting occurred to gather input from stakeholders and the research team members on preliminary findings to provide context and thoughts to inform the potential implications from the review. Participants were notified of both consultation meetings one week prior by email.

## 3 RESULTS

**FIGURE 1:**
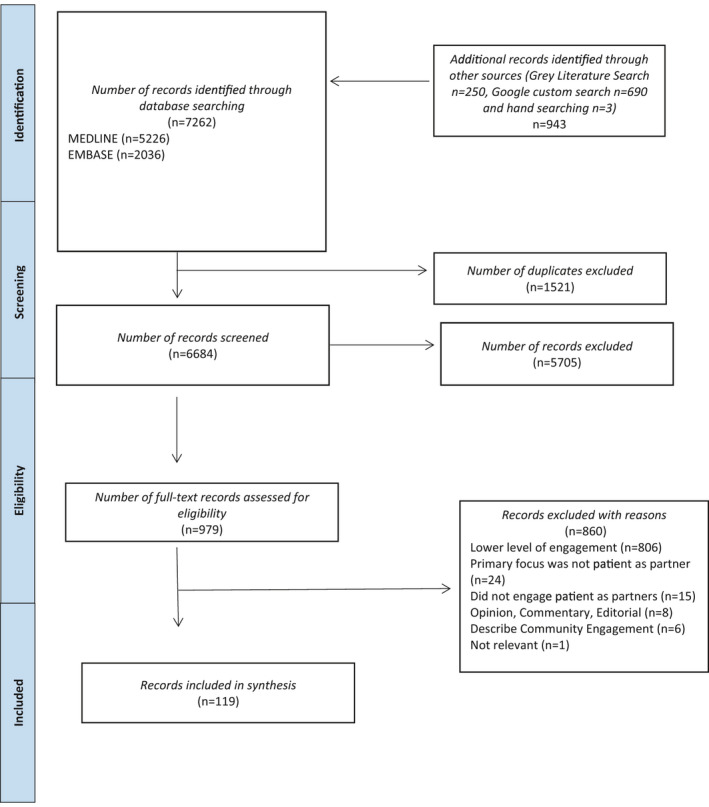
PRISMA flow diagram

After duplicates were removed (n = 1521), 6684 records were screened, and 979 records were selected for full‐text review. A total of 78 academic/peer‐reviewed articles^10^, ^27^, ^28^, ^29^, ^30^, ^31^, ^32^, ^33^, ^34^, ^35^, ^36^, ^37^, ^38^, ^39^, ^40^, ^41^, ^42^, ^43^, ^44^, ^45^, ^46^, ^47^, ^48^, ^49^, ^50^, ^51^, ^52^, ^53^, ^54^, ^55^, ^56^, ^57^, ^58^, ^59^, ^60^, ^61^, ^62^, ^63^, ^64^, ^65^, ^66^, ^67^, ^68^, ^69^, ^70^, ^71^, ^72^, ^73^, ^74^, ^75^, ^76^, ^77^, ^78^, ^79^, ^80^, ^81^, ^82^, ^83^, ^84^, ^85^, ^86^, ^87^, ^88^, ^89^, ^90^, ^91^, ^92^, ^93^, ^94^, ^95^, ^96^, ^97^, ^98^, ^99^, ^100^, ^101^, ^102^, ^103^ and 41 grey literature items^104^, ^105^, ^106^, ^107^, ^108^, ^109^, ^110^, ^111^, ^112^, ^113^, ^114^, ^115^, ^116^, ^117^, ^118^, ^119^, ^120^, ^121^, ^122^, ^123^, ^124^, ^125^, ^126^, ^127^, ^128^, ^129^, ^130^, ^131^, ^132^, ^133^, ^134^, ^135^, ^136^, ^137^, ^138^, ^139^, ^140^, ^141^, ^142^, ^143^, ^144^ were included (Figure 1).

### 3.1 Characteristics of included records

**FIGURE 2:**
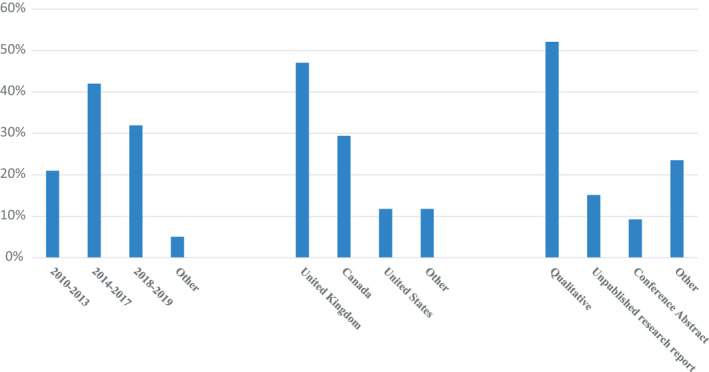
Summary of included record characteristics

Figure 2 shows that the majority of the 119 included records were published in the United Kingdom (n = 56, 47%), followed by Canada (n = 35, 29%) and the United States (n = 14, 12%). The majority of the included records (n = 62, 52%) were published after 2016. The majority of research designs were reported as qualitative (n = 62, 52%), with case studies as the most common (n = 29, 24%), followed by unpublished research reports (n = 18, 15%) resulting from a programme called Patient and Community Engagement Researcher (PaCER).^145^ Table 1 shows the majority of the included records are examples of patients who assume roles as patient partners, (n = 61, 51%) with the most common occurring at the “Collaborate” level (n = 60, 50%). Patient partner characteristics such as age, gender and ethnicity were infrequently reported. See Appendix S5 for a detailed patient partner data including from included studies.

**TABLE 1:** Summary of patient role by level of engagement

Patient role	Level of engagement (# of included records)						
	Involve	Reference	Collaborate	Reference	Lead/support	Reference	Total
Member of research team	9	35, 42, 43, 46, 47, 49, 50, 70, 96	30	10, 30, 32, 34, 37, 38, 41, 44, 48, 51, 53, 58, 60, 62, 64, 66, 67, 68, 69, 71, 73, 74, 75, 77, 78, 80, 81, 94, 101, 128	22	27, 28, 54, 55, 99, 100, 112, 115, 118, 121, 123, 124, 125, 127, 129, 130, 131, 132, 135, 139, 140	61
Member of advisory group	18	33, 36, 57, 63, 65, 88, 89, 90, 91, 95, 105, 107, 109, 114, 119, 126, 134	22	29, 31, 39, 40, 45, 52, 56, 61, 76, 79, 82, 84, 92, 93, 97, 98, 102, 117, 120, 141, 143, 144	2	108, 137	42
Member of steering committee	2	86, 87	3	59, 72, 106	0		5
Member of working group	2	83, 85	2	116, 136	0		4
Not reported	0		3	110, 111, 113	4	104, 122, 133, 142	7
Total	31		60		28		119

### 3.2 Purpose of involvement

A thematic analysis of the purpose of involvement revealed five themes including Documenting and Advancing PPI, Relevance of Research, Co‐building, Capacity Building and Impact on Research.

The first theme, *Documenting and Advancing PPI*, was described when researchers involved patients as partners to describe the process used with the goal of advancing the science of patient involvement (n = 59, 39%). The next theme, *Relevance of Research* was described when researchers involved individuals to make the research more meaningful to those affected, such as by involving patients in developing the research question (n = 37, 24%). The third theme, *Co‐building*, described when researchers involved individuals in the development of a framework, tool or strategy (n = 28, 18%). The fourth theme, *Capacity Building,* described when opportunities were intentionally created to build the skills, confidence and knowledge of patients (n = 16, 10%). The final theme, *Impact on Research*, described the involvement of individuals in the conceptual aspects of research such as defining and refining research scope and research questions as well as the practical elements such as participant recruitment, writing grant proposals and undertaking research (n = 12, 8%). See Table 2.

**TABLE 2:** Purpose of involvement

Theme	Frequency n (%)	Description
Documenting and advancing PPI	59 (39)	This theme described studies where researchers involved individuals in order to customize the engagement strategies to best support their projects and so to contribute to the evidence base by describing the process used such as Berg et al.^29^ who used flexible methods to capture the knowledge and experience of participants
Relevance of research	37 (24)	This theme described when researchers involved individuals to make the research more meaningful to those affected, such as by involving patients in developing the research question such as Sauers‐Ford (2015) who involved parents in the development of the research project
Co‐building	28 (18)	This theme described when researchers involved individuals in co‐developing a framework, tool or strategy such as Horobin et al.^56^ who involved individuals in co‐designing the research tools including the questionnaires and training sessions
Capacity building	16 (10)	This theme described when researchers created opportunities to build the skills, confidence and knowledge of patients such as Dennehy (2018) who engaged individuals using meetings designed to teach individuals a different part of the research process
Impact on research	12 (8)	This theme described the involvement of individuals in the conceptual aspects of research such as defining and refining research scope and research questions as well as the practical elements such as participant recruitment, writing grant proposals and undertaking specific research tasks such as Banfield et al^27^ who involved patients throughout the research project and continually evaluated the process to ensure the research was relevant to those involved

### 3.3 Reported outcomes

**FIGURE 3:**
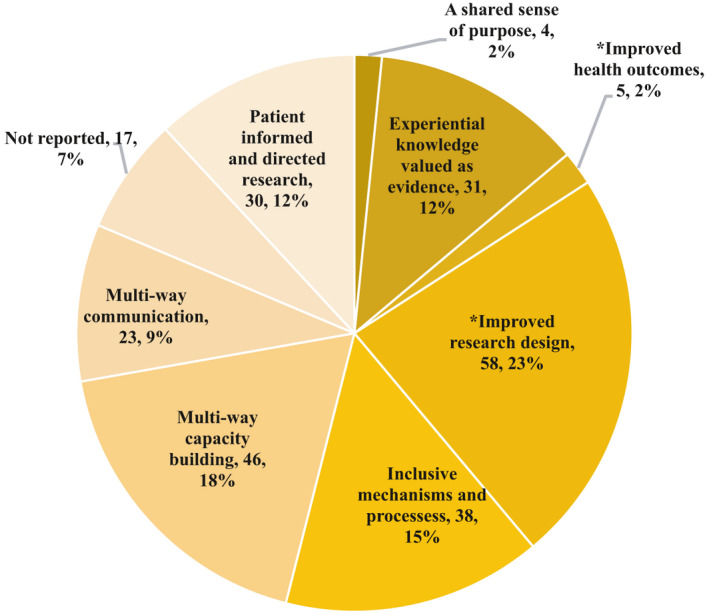
Frequency of study outcome. *Additional outcome categories not currently defined by the CIHR Strategy for Patient Oriented Research – patient engagement framework^7^

Using the CIHR strategy for patient‐oriented research patient engagement strategy as a lens, outcomes were categorized according to one of the six components of successful patient engagement. These included the following: (a) Multi‐way Capacity Building (n = 25, 18%); (b) Inclusive Mechanisms and Processes (n = 19, 14%); (c) Multi‐way Communication and Collaboration (n = 18, 13%); (d) Patient‐informed and Directed Research (n = 7, 5%); (e) Experiential Knowledge is Valued as Evidence (n = 6, 4%); and (f) Shared Sense of Purpose, (n = 3, 2%). During analysis, two additional outcome categories were identified deductively: Improved Research Design, (n = 54; 39%); and Improved Health Outcomes (n = 2; 1%). See Figure 3.

### 3.4 Type of strategies used to support patient partner engagement

Included studies reported using multiple engagement strategies to support patient involvement. Using the word frequency query in NVIVO 12, the most common strategies used to support partner engagement were meetings (n = 37, 31%), followed by research activities where patients were involved in conducting or analysing data such as during focus groups (n = 29, 25%) and interviewing (n = 22, 19%). Regular communication strategies such as email (n = 8, 7%) and teleconferences (n = 8, 7%) were also used (Table 3).

**TABLE 3:** Strategies used to support patient partner engagement

Strategy	Frequency (%)
Meetings	37 (31)
Focus groups	29 (25)
Interviews	22 (19)
Workshops	10 (8)
Email	8 (7)
Teleconference	8 (7)
Other research activities	4 (3)

## 4 DISCUSSION

Acknowledging a newly defined role for patients in health research, we undertook this scoping review to explore the role of patient as research partner. From 6684 academic and grey literature sources, we identified 119 records for inclusion. Performing thematic analysis, we revealed 5 themes for the purpose of engagement: Capacity Building; Impact on Research; Relevance of Research; Documenting and Advancing PPI; and Co‐building. We identified four classifications of the patient partner role including as members of research teams; advisory groups; steering committees; and working groups. We discovered the most commonly reported research designs were qualitative and the types of strategies used to support patient partners during engagement were consistent with activities researchers would use to support other members of the research team. Finally, using the CIHR strategy for patient‐oriented research patient engagement strategy as a lens, we categorized study outcomes into one of the six components of successful patient engagement and identified two additional categories identified during analysis including (a) Multi‐way Capacity Building; (b) Inclusive Mechanisms and Processes; (c) Multi‐way Communication and Collaboration; (d) Patient‐informed and Directed Research; (e) Experiential Knowledge is Valued as Evidence; (f) A Shared Sense of Purpose; (g) Improved Research Design; and, (h) Improved Health Outcomes.

Our findings build on a recent scoping review by Bird, which aimed to understand the impact of patient partnerships on research outcomes, identifying 14 studies which highlight how chronically ill patient partners were involved in research activities. The findings from Bird's review highlight critical barriers and facilitators for researchers seeking to undertake patient partnerships including power imbalances between patient partners and researchers.^1^ Although Bird classified patients into one of the levels as defined by the Patient and Researcher Engagement framework,^15^ we classified studies according to the “higher” levels of engagement (collaborate, involve and lead/support) a decision supported by the literature.^15^, ^16^ We recognize this could imply a hierarchy between the levels of engagement; however, we did not find a notable difference in the study outcomes or type of engagement activities outlined between these three levels. This is a novel finding because often the argument is made that in order to have the greatest impact on a study, patients should assume greater responsibility as co‐researchers. While categorizing study outcomes, two additional outcomes, not described by the CIHR Strategy for Patient Oriented Research, were discovered, Improved Research Design and Improved Health Outcomes. These additional categories suggest the desired outcomes of the CIHR patient engagement framework could be expanded. Our findings also provide evidence of meaningful involvement, suggesting possible characteristics for engagement best practice considerations, described as researchers working alongside patient partners as members of the research team or as an advisory group/committee member, having a clear and mutually agreed upon purpose of the engagement, supported by a number of engagement activities, such as meetings. These findings are supported by Greenhalgh et al, who assert a single, “one‐size‐fits‐all framework” may be less useful than a range of co‐designed activities.^146^

Furthermore, we found that the majority of the studies, regardless of the level of engagement as defined by the higher levels of the spectrum (involve, collaborate or lead/support), reported successful engagement. This change is in part because of the evolution of dedicated infrastructure and resources from governments, funders and partner organizations to prioritize patient engagement or involvement in research. Staley argues that a gap in understanding still remains in how the impacts or outcomes of patient engagement are achieved and suggests the solution is in providing more detailed accounts of patient involvement.^147^ Given many of the included records infrequently reported key characteristics such as age and ethnicity, we suggest researchers go even further to report patient partner characteristics so comparisons can be made.

Additionally, national priorities and additional resources have precipitated further changes, with a surge of records on PPI since 2016 identified by this review (n = 62, 52%), primarily from the United Kingdom, Canada and the United States (47%, 29% and 12%, respectively). In contrast, patient involvement in research in low‐ and middle‐income countries (LMIC) is uncommon.^148^ This may be due to lack of health infrastructure, socioeconomic status, cultural stigma and uncertain roles, but have the potential to improve with international‐focused collaboration.^148^ However, we recognize that the majority of studies found were qualitative, specifically case studies or mixed methods in design. We recognize that patient engagement approaches in clinical trials^149^, ^150^ and data‐intensive health research^151^ require additional investment in training and capacity building for both patients and researchers.

### 4.1 Strengths and limitations

This study has strengths and limitations. Due to the comprehensive scope of the search, the volume of studies required us to limit to two academic databases, limit the publication dates of the included studies and limit our search to English language peer‐reviewed publications. These decisions were made to maintain the feasibility of the project. These limitations were balanced with a robust grey literature and hand‐searching strategy. Despite this, it is possible that a search without these imposed limitations may have yielded additional studies of relevance to this review. We identified the “higher” levels of engagement as a source of examples to describe patient as research partners. We believe our findings support this decision but suggest additional research be undertaken to further understand the similarities and/or differences between roles providing greater involvement, increased sharing of power and increased responsibilities and those that do not.^16^ When assigning studies to one of the six levels of engagement, as described by the Patient and Researcher Engagement spectrum,^15^ we assigned the level of engagement based on what was reported by the manuscript authors and acknowledge it is possible some studies were incorrectly assigned. We believe the duplicate review of each study and the addition of a third reviewer when consensus could not be reached minimized potential errors. Further, the patient partner‐led grey literature search and data extraction may be perceived as design bias by some. However, we feel that the robust methodological processes we developed to conduct this review minimized any potential for bias, while supporting greater understanding and confidence among the patient partners.

## 5 CONCLUSION

While significant research exists that highlights how researchers are involving patients in health research, the engagement of patient as research partner is not well described or understood. Our findings suggest, with much research focused on the mechanisms of how and theoretical frameworks of why to engage patients, the linkage between purpose of engagement and study outcomes are evident. The data set also enabled a greater understanding of the role of patient as partner in health research. Creating opportunities for the involvement of patients as partners, in all aspects of research, and across research designs, helps researchers and patients in building a critical mass for change on an individual and an organizational level.

## CONFLICT OF INTERESTS

The authors declare that they have no conflict of interest.

## AUTHOR CONTRIBUTIONS

TLM had significant involvement in the design, acquisition, analysis and interpretation of data. FC, TW and MS provided guidance in the overall design and delivery of the research. MS, AG, CM, JR and TLM were involved in the acquisition and final analysis. All authors provided revisions and the final approval to be published. All the named authors agree to take accountability for the integrity and accuracy of the work and have read and approved the final manuscript.

## Supporting information

Supplementary Material

Supplementary Material

Supplementary Material

Supplementary Material

Supplementary Material

## Data Availability

All data generated or analysed during this study were included in this published article and/or its supplementary materials.
