# Supplementary material for: Patients as partners in health research: A scoping review
Source: Health Expect. 2021 Jun 21;24(4):1378–90. doi: 10.1111/hex.13272 (PMC8369093; doi:10.1111/hex.13272)
Supplement: Supplementary file 1 — Supplementary Material [file HEX-24-1378-s004.docx]

Appendix 1: MedLINE Search Strategy

1. exp Community Participation/ or Patient Participation/
2. ((carer* or caregiver* or community or consumer* or family or families or patient* or public) adj3 (incorporat* or involv* or integrat* or participation or engag*)).tw,kw.
3. ((carer* or caregiver* or community or consumer* or family or families or patient* or public) adj3 (co-creat* or cocreat* or coproduc* or co-produc* or codesign* or co-design* or governance)).tw,kw.
4. (Patient adj (research or partner)).tw,kw.
5. 1 or 2 or 3 or 4
6. exp Health Services Research/ or (health adj2 research).tw,kw. or (health adj2 decision making).tw,kw.
7. limit 7 to yr="2010 - 2019"
8. limit 8 to english
9. limit 9 to (conference abstract or editorial or letter)
10. 9 not 10
