# Supplementary material for: Patients as partners in health research: A scoping review
Source: Health Expect. 2021 Jun 21;24(4):1378–90. doi: 10.1111/hex.13272 (PMC8369093; doi:10.1111/hex.13272)
Supplement: Supplementary file 2 — Supplementary Material [file HEX-24-1378-s003.docx]

**Search terms**

| **Key Concept** | **Search Terms** |
| --- | --- |
| Patient Engagement | patient engagement  patient partner  patient involvement  consumer engagement  consumer involvement  community engagement |

**Decision-Tree for How to Search Key Websites**

*Don’t forget to think about spectrum of engagement

Y

Y

Y

N

N

New website

Is there a search option? A symbol of a magnifying glass?

Type in search terms. *Explore & report findings in data sheet

Is there a resources tab?

Type in combination of search terms. *Explore & report findings in data sheet

Report these findings on the data sheet

| **ID** | **Website** | **Findings (Paste URLs). Entering RETURN on your keyboard in this cell will give you more space** | **Comments (if any)** |
| --- | --- | --- | --- |
|  | <https://ossu.ca>  searched; nothing found  not searched; not relevant  searched; results found  results may be of little importance – requires discussion |  |  |
|  | <http://www.ochsu.ca/en/OCHSU>  searched; nothing found  not searched; not relevant  searched; results found  results may be of little importance – requires discussion |  |  |
|  | <http://www.spor-maritime-srap.ca/patients-and-public>  searched; nothing found  not searched; not relevant  searched; results found  results may be of little importance – requires discussion |  |  |
|  | <http://www.nlsupport.ca/training.aspx>  searched; nothing found  not searched; not relevant  searched; results found  results may be of little importance requires discussion |  |  |
|  | [https://chimb.ca/leadership](https://chimb.ca/leadershipdevelopmentandtraining)  [developmentandtraining](https://chimb.ca/leadershipdevelopmentandtraining)  searched; nothing found  not searched; not relevant  searched; results found  results may be of little importance – requires discussion |  |  |
|  | <https://ceppp.ca/en/resources/>  searched; nothing found  not searched; not relevant  searched; results found  results may be of little importance – requires discussion |  |  |
|  | <https://spor.albertainnovates.ca/the-alberta-spor-support-unit/patient-engagement-platform/resources/>  searched; nothing found  not searched; not relevant  searched; results found  results may be of little importance – requires discussion |  |  |
|  | <https://bcsupportunit.ca/resources>  searched; nothing found  not searched; not relevant  searched; results found  results may be of little importance – requires discussion |  |  |
|  | <https://www.scpor.ca/>  searched; nothing found  not searched; not relevant  searched; results found  results may be of little importance – requires discussion |  |  |
|  | <https://nwtspor.ca/>  searched; nothing found  not searched; not relevant  searched; results found  results may be of little importance – requires discussion |  |  |
|  | <https://www.nbpihcinetwork.ca/>  searched; nothing found  not searched; not relevant  searched; results found  results may be of little importance – requires discussion |  |  |
|  | <http://alberta-spor-pihcin.com/>  searched; nothing found  not searched; not relevant  searched; results found  results may be of little importance – requires discussion |  |  |
|  | <http://www.ichr.ca/research/the-northwest-territories-spor-network/>  searched; nothing found  not searched; not relevant  searched; results found  results may be of little importance – requires discussion |  |  |
|  | <https://spor-bcphcrn.ca/>  searched; nothing found  not searched; not relevant  searched; results found  results may be of little importance – requires discussion |  |  |
|  | <https://manitoba-pihcinet.com/>  searched; nothing found  not searched; not relevant  searched; results found  results may be of little importance – requires discussion |  |  |
|  | <https://mun.yaffle.ca/networks/3135>  searched; nothing found  not searched; not relevant  searched; results found  results may be of little importance – requires discussion |  |  |
|  | <https://www.beaccon.ca/>  searched; nothing found  not searched; not relevant  searched; results found  results may be of little importance – requires discussion |  |  |
|  | <http://projects.upei.ca/spor-pei/>  searched; nothing found  not searched; not relevant  searched; results found  results may be of little importance – requires discussion |  |  |
|  | <http://www.frqs.gouv.qc.ca/en/la-recherche/la-recherche-financee-par-le-frqs/centres-groupes-et-reseaux/groupe?id=pq4r2dcu1401199512271&>  searched; nothing found  not searched; not relevant  searched; results found  results may be of little importance – requires discussion |  |  |
|  | <https://accessopenminds.ca/>  searched; nothing found  not searched; not relevant  searched; results found  results may be of little importance – requires discussion |  |  |
|  | <https://www.child-bright.ca/>  searched; nothing found  not searched; not relevant  searched; results found  results may be of little importance – requires discussion |  |  |
|  | <https://www.cansolveckd.ca/>  searched; nothing found  not searched; not relevant  searched; results found  results may be of little importance – requires discussion |  |  |
|  | <http://imaginespor.com/>  searched; nothing found  not searched; not relevant  searched; results found  results may be of little importance – requires discussion |  |  |
|  | <https://diabetesaction.ca/>  searched; nothing found  not searched; not relevant  searched; results found  results may be of little importance – requires discussion |  |  |
|  | <https://cpn.mcmaster.ca/>  searched; nothing found  not searched; not relevant  searched; results found  results may be of little importance – requires discussion |  |  |
|  | <http://ktcanada.net/>  searched; nothing found  not searched; not relevant  searched; results found  results may be of little importance – requires discussion |  |  |
|  | <https://ebm-tools.knowledgetranslation.net/>  searched; nothing found  not searched; not relevant  searched; results found  results may be of little importance – requires discussion |  |  |
|  | <https://ktalberta.ca/>  searched; nothing found  not searched; not relevant  searched; results found  results may be of little importance – requires discussion |  |  |
|  | <http://www.ohri.ca/ksgroup/Default.aspx>  searched; nothing found  not searched; not relevant  searched; results found  results may be of little importance – requires discussion |  |  |
|  | <https://www.hqca.ca/about/>  searched; nothing found  not searched; not relevant  searched; results found  results may be of little importance – requires discussion |  |  |
|  | <https://www.hqontario.ca/about-us>  searched; nothing found  not searched; not relevant  searched; results found  results may be of little importance – requires discussion |  |  |
|  | <https://bcpsqc.ca/>  searched; nothing found  not searched; not relevant  searched; results found  results may be of little importance – requires discussion |  |  |
|  | <http://www.hqc.sk.ca>  searched; nothing found  not searched; not relevant  searched; results found  results may be of little importance – requires discussion |  |  |
|  | <http://mips.ca>  searched; nothing found  not searched; not relevant  searched; results found  results may be of little importance – requires discussion |  |  |
|  | <http://www.csbe.gouv.qc.ca/en/home.html>  searched; nothing found  not searched; not relevant  searched; results found  results may be of little importance – requires discussion |  |  |
|  | <http://www.nbhc.ca>  searched; nothing found  not searched; not relevant  searched; results found  results may be of little importance – requires discussion |  |  |
|  | <https://www.accreditation.ca>  searched; nothing found  not searched; not relevant  searched; results found  results may be of little importance – requires discussion |  |  |
|  | <https://www.invo.org.uk/>  searched; nothing found  not searched; not relevant  searched; results found  results may be of little importance – requires discussion |  |  |
|  | <http://www.healthcareimprovementscotland.org/>  searched; nothing found  not searched; not relevant  searched; results found  results may be of little importance – requires discussion |  |  |
|  | <https://www.nice.org.uk/about>  searched; nothing found  not searched; not relevant  searched; results found  results may be of little importance – requires discussion |  |  |
|  | <http://www.io.nihr.ac.uk/what-we-do/>  searched; nothing found  not searched; not relevant  searched; results found  results may be of little importance – requires discussion |  |  |
|  | <https://www.pcori.org/>  searched; nothing found  not searched; not relevant  searched; results found  results may be of little importance – requires discussion |  |  |
|  | <https://cihr-irsc.gc.ca/e/41204.html>  searched; nothing found  not searched; not relevant  searched; results found  results may be of little importance – requires discussion |  |  |
|  | <https://chf.org.au/>  searched; nothing found  not searched; not relevant  searched; results found  results may be of little importance – requires discussion  patient engagement  consumer engagement  consumer involvement |  |  |
|  | <https://www.cfhi-fcass.ca/>  searched; nothing found  not searched; not relevant  searched; results found  results may be of little importance – requires discussion |  |  |
|  | <https://imaginecitizens.ca/>  searched; nothing found  not searched; not relevant  searched; results found  results may be of little importance – requires discussion |  |  |
|  | <https://cadth.ca>  searched; nothing found  not searched; not relevant  searched; results found  results may be of little importance – requires discussion |  |  |
|  | <http://www.opengrey.eu>  searched; nothing found  not searched; not relevant  searched; results found  results may be of little importance – requires discussion |  |  |
|  | <https://library.ucalgary.ca/c.php?g=707850&p=5039229>  searched; nothing found  not searched; not relevant  searched; results found  results may be of little importance – requires discussion |  |  |
|  | <https://www.patientsafetyinstitute.ca/>  searched; nothing found  not searched; not relevant  searched; results found  results may be of little importance – requires discussion |  |  |
|  | <https://www.healthaffairs.org/>  searched; nothing found  not searched; not relevant  searched; results found  results may be of little importance – requires discussion |  |  |
|  | <https://www.evidence.nhs.uk>  searched; nothing found  not searched; not relevant  searched; results found  results may be of little importance – requires discussion |  |  |
|  | <https://www.picker.org>  searched; nothing found  not searched; not relevant  searched; results found  results may be of little importance – requires discussion |  |  |
|  | <https://www.safetyandquality.gov.au>  searched; nothing found  not searched; not relevant  searched; results found  results may be of little importance – requires discussion |  |  |
|  | <https://www.nhmrc.gov.au>  searched; nothing found  not searched; not relevant  searched; results found  results may be of little importance – requires discussion |  |  |
|  | <https://www.hqsc.govt.nz>  searched; nothing found  not searched; not relevant  searched; results found  results may be of little importance – requires discussion |  |  |
|  | <https://partnershipforpatients.cms.gov>  searched; nothing found  not searched; not relevant  searched; results found  results may be of little importance – requires discussion |  |  |
|  | <https://www.fda.gov>  searched; nothing found  not searched; not relevant  searched; results found  results may be of little importance – requires discussion |  |  |
