# Supplementary material for: Patients as partners in health research: A scoping review
Source: Health Expect. 2021 Jun 21;24(4):1378–90. doi: 10.1111/hex.13272 (PMC8369093; doi:10.1111/hex.13272)
Supplement: Supplementary file 3 — Supplementary Material [file HEX-24-1378-s001.docx]

Appendix 3: Custom Google Search Terms

| **Key Concept** | **Search Terms** | | |
| --- | --- | --- | --- |
| Patient Engagement | ‘community participation’  ‘community incorporation’  ‘community involvement’  ‘community integration’  ‘community engagement’  ‘community cocreation’  ‘community co-creation’  ‘community coproduction’  ‘community co-production’  ‘community codesign’  ‘community co-design’  ‘community governance’ | ‘patient participation’  ‘patient incorporation’  ‘patient involvement’  ‘patient integration’  ‘patient engagement’  ‘patient cocreation’  ‘patient co-creation’  ‘patient coproduction’  ‘patient co-production’  ‘patient codesign’  ‘patient co-design’  ‘patient governance’  ‘patient partner’ | ‘public participation’  ‘public incorporation’  ‘public involvement’  ‘public integration’  ‘public engagement’  ‘public cocreation’  ‘public co-creation’  ‘public coproduction’  ‘public co-production’  ‘public codesign’  ‘public co-design’  ‘public governance’ |
|  | ‘carer participation’  ‘carer incorporation’  ‘carer involvement’  ‘carer integration’  ‘carer engagement’  ‘carer cocreation’  ‘carer co-creation’  ‘carer coproduction’  ‘carer co-production’  ‘carer codesign’  ‘carer co-design’  ‘carer governance’ | ‘caregiver participation’  ‘caregiver incorporation’  ‘caregiver involvement’  ‘caregiver integration’  ‘caregiver engagement’  ‘caregiver cocreation’  ‘caregiver co-creation’  ‘caregiver coproduction’  ‘caregiver co-production’  ‘caregiver codesign’  ‘caregiver co-design’  ‘caregiver governance’ | ‘family participation’  ‘family incorporation’  ‘family involvement’  ‘family integration’  ‘family engagement’  ‘family cocreation’  ‘family co-creation’  ‘family coproduction’  ‘family co-production’  ‘family codesign’  ‘family co-design’  ‘family governance’ |
|  | ‘consumer participation’  ‘consumer incorporation’  ‘consumer involvement’  ‘consumer integration’  ‘consumer engagement’  ‘consumer cocreation’  ‘consumer co-creation’  ‘consumer coproduction’  ‘consumer co-production’  ‘consumer codesign’  ‘consumer co-design’  ‘consumer governance’ | ‘families participation’  ‘families incorporation’  ‘families involvement’  ‘families integration’  ‘families engagement’  ‘families cocreation’  ‘families co-creation’  ‘families coproduction’  ‘families co-production’  ‘families codesign’  ‘families co-design’  ‘families governance’ |  |
