# Supplementary material for: Patients as partners in health research: A scoping review
Source: Health Expect. 2021 Jun 21;24(4):1378–90. doi: 10.1111/hex.13272 (PMC8369093; doi:10.1111/hex.13272)
Supplement: Supplementary file 5 — Supplementary Material [file HEX-24-1378-s002.docx]

Appendix 5: Patient Partner Data from Included Studies

| Ref | Author | Year | Country | | Design | | Level* | | Purpose of Engagement (Theme) | | Strategies to Support Partner Engagement | | Description of Patient Partner Role | | Study Outcomes (Categories) | |  |
| --- | --- | --- | --- | --- | --- | --- | --- | --- | --- | --- | --- | --- | --- | --- | --- | --- | --- |
| 12 | McCarron | 2019 | Canada | | Quantitative | | C | | • Documenting and advancing PPI | | Patients acted as co-investigators and were involved in the in the design and delivery of the research, from project inception to final analysis and write‐up. | | Member of research team | | • NR | |  |
| 22 | Banfield | 2018 | Australia | | Qualitative | | L | | • Capacity Building • Relevance of research  • Documenting and advancing PPI • Impact on the research process | | Face-to-face meetings; out-of-work sessions; all stages were continually evaluated to ensure research was relevant to those involved. | | Member of research team | | • Patient-informed and directed research • Inclusive Mechanisms and Processes • Improved research design • Multi-way Capacity Building  • Multi-way Communication and Collaboration • Experiential knowledge valued as evidence | |  |
| 23 | Bates | 2018 | Africa | | Qualitative | | L | | • Relevance of research | | Met twice a week over a 4-week period. Attended nine half-day group sessions over a 4-month period and a final advocacy event | | Member of research team | | • Inclusive Mechanisms and Processes • Multi-way Capacity Building  • Multi-way Communication and Collaboration • Improved research design | |  |
| 24 | Berg | 2013 | Sweden | | Qualitative | | C | | • Documenting and advancing PPI • Co-building | | Participatory design, using flexible methods, was used to capture the knowledge and experience of participants. Partners attended a half day workshop to test and evaluate the protype. | | Member of advisory group | | • Experiential knowledge valued as evidence | |  |
| 25 | Best | 2017 | United Kingdom | | Qualitative | | C | | • Co-building | | Partners involved in data analysis to sort into piles based on their perception of related thematic content. | | Member of research team | | • Improved research design • Multi-way Communication and Collaboration • Inclusive Mechanisms and Processes • Improved research design | |  |
| 26 | Blomqvist | 2010 | Sweden | | Qualitative | | C | | • Relevance of research  • Co-building | | Participatory action approach was used to provide flexibility; a number of dialogue events in in the form of focus groups were held. | | Member of advisory group | | • Improved research design | |  |
| 27 | Boyer | 2018 | United States | | Mixed Methods | | C | | • Documenting and advancing PPI • Co-building | | Stakeholders were important research team members on oversight and advisory committees; through interviews and surveys. | | Member of research team | | • Improved research design | |  |
| 28 | Brach | 2016 | United States | | Quantitative | | I | | • Relevance of research  • Documenting and advancing PPI | | Participant stakeholders were involved in the preparation of research activities and focus groups to help develop the intervention. They are also involved in the execution and translation phases as members of our Community Advisory Boards. | | Member of advisory group | | • Multi-way Communication and Collaboration • Inclusive Mechanisms and Processes | |  |
| 29 | Brainard | 2017 | United Kingdom | | Qualitative | | C | | • Relevance of research  • Impact on the research process | | Participated in brainstorming and rapid testing process, invited to give feedback at both meetings and via email between meetings. | | Member of research team | | • Improved research design | |  |
| 30 | Brear | 2019 | Australia | | Qualitative | | I | | • Documenting and advancing PPI | | Participated in all aspects; dialogic learning workshops. These were the setting in which the coresearchers learnt about research design, ethics and data collection, and qualitative data analysis. They also codesigned the methodology, methods, instruments, and ethical procedures; participated in data analysis and interpretation; planned actions based on the results; and member checked my findings. | | Member of research team | | • Improved research design | |  |
| 31 | Brereton | 2017 | United Kingdom | | Qualitative | | I | | • Documenting and advancing PPI | | Group and individual meetings; lay and professional palliative care stakeholders in seven European countries acted as ‘advisors’ to inform project development from an early stage. As ‘advisors’, stakeholders provided information or data that informed researchers’ decision making in the project. Local co-ordinators were established in each country and were given a guide to assist in establishing some consistency in planning stakeholder involvement, including example documentation (e.g. information sheets, consent forms). | | Member of advisory group | | • Multi-way Communication and Collaboration • Inclusive Mechanisms and Processes • Improved research design | |  |
| 32 | Callander | 2011 | Australia | | Qualitative | | C | | • Documenting and advancing PPI | | Two teams met twice for 1.5 hours to discuss their original perceptions and expectations of the project, the challenges or barriers they faced and how these were overcome. | | Member of research team | | • Multi-way Capacity Building | |  |
| 33 | Carr | 2019 | Canada | | Qualitative | | C | | • Co-building | | Bi-monthly one-hour telephone conference calls with follow up calls and email contact between meetings, as required. | | Member of research team | | • Improved research design | |  |
| 34 | Chiu | 2013 | Canada | | Qualitative, Case Study | | C | | • Documenting and advancing PPI | | Mixed method engagement; Qualitative interviews and survey | | Member of advisory group | | • Improved research design | |  |
| 35 | Clarke | 2018 | United Kingdom | | Qualitative | | C | | • Documenting and advancing PPI | | Analyzed data in workshops with people living with dementia; The design involved cycles of presenting, interpreting, representing and reinterpreting the data, and findings between multiple stakeholders. | | Member of advisory group | | • Multi-way Capacity Building  • Multi-way Communication and Collaboration • Patient-informed and directed research | |  |
| 36 | Concannon | 2014 | United States | | Review | | C | | • Documenting and advancing PPI | | Worked with stakeholders before, during and after the review was conducted to define the primary and key research questions; conduct the literature search; screen titles, abstracts and articles; abstract data from the articles; and analyze the data. | | Member of research team | | • A shared sense of purpose | |  |
| 37 | Coon | 2016 | United Kingdom | | Review | | I | | • Documenting and advancing PPI | | End-users were involved in all stages of the project, both as authors and as members of an advisory group. In addition, several events were held with groups of relevant end-users during the project. | | Member of research team | | • Multi-way Capacity Building  • Improved research design | |  |
| 38 | Costello | 2019 | United Kingdom | | Qualitative | | involve | | • Documenting and advancing PPI | | Used co-designed approach with patients or caregivers involved at every stage: concept, design, format, choice of seminar location, choice of research topics, target age range, review of programme, review of presentation, feedback, and writing and reviewing the manuscript. | | Member of research team | | • NR | |  |
| 39 | Dennehy | 2018 | United Kingdom | | Qualitative | | C | | • Capacity Building | | Engaged via meetings. Each of the meetings aimed to teach them different parts of the research process. Icebreakers were used, capacity building was important to engage with the research and issues surrounding cyberbullying as well as capture their views on cyberbullying. | | Member of research team | | • Multi-way Communication and Collaboration • Inclusive Mechanisms and Processes • Multi-way Capacity Building  • Patient-informed and directed research • Improved research design | |  |
| 40 | Dovey-Pearce | 2019 | United Kingdom | | Qualitative | | C | | • Relevance of research | | Flexible approaches were used including focus groups and interviews. | | Member of advisory group | | • Multi-way Communication and Collaboration • Multi-way Capacity Building  • Multi-way Communication and Collaboration • Inclusive Mechanisms and Processes • Improved research design | |  |
| 41 | Elliot | 2018 | Canada | | Qualitative | | I | | • Documenting and advancing PPI | | Individuals were engaged in different types of activities including involvement in the priority-setting workshop, wiki online tool, and/or steering committee. | | Member of research team | | • Multi-way Communication and Collaboration • Inclusive Mechanisms and Processes | |  |
| 42 | Garwick | 2010 | United States | | Qualitative | | I | | • Documenting and advancing PPI | | Project began by identifying a parent advisory group providing guidance throughout the project. Project had three phases; Phase 1 - Focus groups lasting 1.5 hours, Phase 2 - Web-based asthma resources were identified and evaluated; and Phase 3 - Parents were invited to an action plan meeting where they reviewed and critiques the content and format of the preliminary website. | | Member of research team | | • Multi-way Capacity Building | |  |
| 43 | Evans | 2019 | United Kingdom | | Qualitative | | C | | • Documenting and advancing PPI • Co-building | | Individuals with experience of chronic conditions, as patients and carers, were supported to develop and implement an involvement model. Two workshops, using a modified Normative Group Technique, regular email updates and quarterly meetings held in person or Skype over 8-year study period. | | Member of research team | | • Improved research design • Multi-way Capacity Building  • Multi-way Communication and Collaboration • Inclusive Mechanisms and Processes • Improved research design • Patient-informed and directed research | |  |
| 44 | Gibson | 2017 | United Kingdom | | Qualitative | | I | | • Documenting and advancing PPI | | Three workshops with different PPI groups were conducted in which participants were invited to map their PPI experiences on wall charts representing the four dimensions of the established framework. | | Member of research team | | • Experiential knowledge valued as evidence | |  |
| 45 | Lowes | 2011 | United Kingdom | | Qualitative, Case Study | | I | | • Documenting and advancing PPI | | Meetings over 3 full days over the course of 10 months with independent facilitator to run the meetings. | | Member of research team | | • Improved research design | |  |
| 46 | Gillard | 2012 | United Kingdom | | Qualitative | | C | | • Documenting and advancing PPI | | Frequent, usually weekly, meetings were held in each study site where the service user and carer researchers were supervised by the site lead. Five whole-team meetings took place throughout the study, with communications and discussions taking place by teleconference and email between meetings Team collaboration, leading the project. | | Member of research team | | • Multi-way Communication and Collaboration | |  |
| 47 | Hamilton | 2018 | Canada | | Qualitative | | C | | • Capacity Building • Co-building | | Patient researchers were interviewed about their experience and views working on a research team | | Member of advisory group | | • NR | |  |
| 48 | Hamilton | 2018 | Canada | | Mixed Methods | | C | | • Relevance of research | | Surveys, interviews, patients as part of the research team (data validation, writing the manuscript etc. Research conducted over three phases. | | Member of research team | | • Improved research design | |  |
| 49 | Hofmann | 2013 | United Kingdom | | Qualitative | | L | | • Co-building | | Focus groups, data analysis, and recruitment of participants. | | Member of research team | | • Inclusive Mechanisms and Processes | |  |
| 50 | Holmes | 2019 | United Kingdom | | Qualitative, Case Study | | L | | • Impact on the research process | | Using an interactive 'cycle' of engagement, involvement and research; delivering sessions, focus groups, and involved communication. | | Member of research team | | • Inclusive Mechanisms and Processes • Improved health outcomes  • Multi-way Capacity Building  • Inclusive Mechanisms and Processes | |  |
| 51 | Horobin | 2017 | United Kingdom | | Qualitative, Case Study | | C | | • Co-building | | Meetings, co-designing, questionnaires, and training sessions. | | Member of advisory group | | • Multi-way Capacity Building  • Inclusive Mechanisms and Processes | |  |
| 52 | Hull | 2012 | United Kingdom | | Qualitative, Case Study | | I | | • Documenting and advancing PPI | | Reviewing exercises, surveys and part of an advisory panel. | | Member of advisory group | | • Improved research design | |  |
| 53 | Iliffe | 2013 | United Kingdom | | Qualitative, Case Study | | C | | • Impact on the research process | | Steering committees, focus groups, interviews, and panels | | Member of research team | | • Improved research design • Experiential knowledge valued as evidence | |  |
| 54 | Irving | 2018 | United Kingdom | | Qualitative, Case Study | | C | | • Capacity Building • Relevance of research | | Surveys, events, meetings, steering committee; and the event was co-produced. | | Member of steering committee | | • Improved research design • Improved research design | |  |
| 55 | Jennings | 2018 | United Kingdom | | Review | | C | | • Impact on the research process | | Co-researchers involved in interpreting results and key research activities. | | Member of research team | | • Improved research design | |  |
| 56 | Jorgensen | 2018 | Denmark | | Qualitative | | C | | • Relevance of research | | Provided feedback on proposal, assisted in development of project documents and research tools, acted as peer interviewers during qualitative interviews, participated in data analysis, co-authored journal articles, and were co-presenters at conferences with researchers. | | Member of advisory group | | • Improved research design | |  |
| 57 | Jorgensen | 2018 | Denmark | | Qualitative | | C | | • Documenting and advancing PPI | | Patient representatives involved in all phases of overall project: research proposal (before submission for funding), steering group including two patient representatives, and co-researchers involved in all aspects of the study. Of the 9 co-researchers, 5 became peer interviewers and carried out 1-4 interviews each. Peer interviewers were also involved in the development of the semi-structured interview guide. | | Member of research team | | • Improved research design | |  |
| 58 | Kandiyali | 2019 | United Kingdom | | Qualitative, Case Study | | I | | • Documenting and advancing PPI | | Research Activities: Developed research activities, helped inform content and delivery of intervention and data collection. | | Member of advisory group | | • Improved research design | |  |
| 59 | Keenan | 2019 | United Kingdom | | Qualitative | | C | | • Documenting and advancing PPI | | Participated in training and supported focus group planning, discussion guide refinement, and facilitated/observed groups, and data analysis | | Member of research team | | • Inclusive Mechanisms and Processes | |  |
| 60 | Kim | 2018 | United States | | Qualitative, Case Study | | I | | • Documenting and advancing PPI | | Meetings: Participated in the planning and developing governance activities and refining policies, research management, and participant protection elements of research governance. | | Member of advisory group | | • Patient-informed and directed research | |  |
| 61 | Kristensen | 2018 | | Denmark | | Qualitative, Case Study | | C | | • Relevance of research  • Co-building | | Patient Peer Board (PBG) discussed item relevance, mode of data collection, graphical format of online PROMS, display or results through a series of workshops. Workshop content was directed by steering group (SG), which included patient representatives and final items/modes of delivery/format of PROMs approved by the SG. Consensus recommendations made to incorporate recommendations from both PPB and SG into final clinical product. | | Member of research team | | • Improved research design | |
| 62 | Larkin | 2015 | | United Kingdom | | Qualitative, Case Study | | C | | • Capacity Building • Co-building | | Engagement throughout project including feedback groups; co-designed project; and steering committee. | | Member of research team | | • Improved research design | |
| 63 | Lesse | 2018 | | Canada | | Qualitative | | C | | • Documenting and advancing PPI | | The study was initiated and conducted by patient partners who were involved in all phases of research design, including co-designing study, analysis, and dissemination | | Member of research team | | • NR | |
| 64 | Likumahuwa-Ackman | 2015 | | United States | | Qualitative, Case Study | | C | | • Relevance of research | | Advisory groups, interviews, Think Aloud activities, and acted patient partner as co-investigator. | | Member of research team | | • Improved research design | |
| 65 | Vale | 2012 | | United Kingdom | | Review | | I | | • Documenting and advancing PPI | | Research Involvement in research activities, meetings, writing research materials, and recruitment. | | Member of research team | | • Multi-way Communication and Collaboration | |
| 66 | Malus | 2011 | | Canada | | Mixed Methods | | C | | • Capacity Building | | Flexible Approach: Using a participatory approach, an Interdisciplinary committee composed of patients, staff and researchers (patients formed the majority of members) worked together as the primary decision-making body throughout the project. | | Member of research team | | • Improved research design | |
| 67 | Mann | 2018 | | United Kingdom | | Qualitative, Case Study | | C | | • Documenting and advancing PPI • Impact on the research process | | Advisory group, involved in all phases of research, PPI co-ordinator facilitated communication/engagement/training of co-researchers | | Member of steering committee | | • Multi-way Communication and Collaboration | |
| 68 | Marks | 2018 | | United Kingdom | | Qualitative, Case Study | | C | | • Documenting and advancing PPI | | Participation in various advisory groups, co-research activities ranging from document review, input into protocol, data analysis, to dissemination. | | Member of research team | | • Improved research design • Improved research design | |
| 69 | Mawn | 2015 | | United Kingdom | | Qualitative, Case Study | | C | | • Capacity Building • Impact on the research process | | Youth research group, discussed research directions, provided input, and involved in various parts of the research process. | | Member of research team | | • Inclusive Mechanisms and Processes • Multi-way Capacity Building | |
| 70 | McCarron | 2019 | | Canada | | Review | | C | | • Capacity Building • Documenting and advancing PPI | | Patients acted as co-investigators and were involved in research process including meetings to determine scope of project, data collection, analysis and co-authors on paper. | | Member of research team | | • Multi-way Capacity Building | |
| 71 | Mockford | 2016 | | United Kingdom | | Qualitative, Case Study | | C | | • Documenting and advancing PPI | | Advisory group meetings and responsibilities as co-researchers findings from study design to data analysis. | | Member of advisory group | | • Improved research design • Improved research design | |
| 72 | Moltu | 2012 | | Norway | | Qualitative | | C | | • Capacity Building | | Preliminary analyses were taken back to the partners for discussion, auditing, and reanalysis. | | Member of research team | | • Multi-way Capacity Building  • Patient-informed and directed research | |
| 73 | Nierse | 2011 | | Netherlands | | Qualitative, Case Study | | C | | • Documenting and advancing PPI | | Partners on the research team participated in focus groups, interviews and analysis. | | Member of research team | | • Improved research design • Multi-way Capacity Building | |
| 74 | Nierse | 2012 | | Netherlands | | Qualitative, Case Study | | C | | • Relevance of research  • Documenting and advancing PPI • Co-building | | Partners on the research team participated in focus groups, interviews and analysis. | | Member of advisory group | | • Improved research design • Multi-way Capacity Building  • Multi-way Communication and Collaboration • Improved research design • Inclusive Mechanisms and Processes | |
| 75 | Nowell | 2018 | | United States | | Qualitative, Case Study | | C | | • Documenting and advancing PPI | | Patients acted as patient governors; defining this role and creating an advisory role made up completely of patients to better address the needs of patients; participated in evaluation and other key activities during the start-up phase of establishing this new role. | | Member of research team | | • Improved research design | |
| 76 | O'Donnell | 2019 | | United Kingdom | | Qualitative, Case Study | | C | | • Capacity Building • Documenting and advancing PPI | | Involved throughout including in establishing the research environment; expectations and role clarity; support for participation and inclusive representation and; commitment to the value of co-learning. | | Member of research team | | • Multi-way Capacity Building | |
| 77 | O'Gorman | 2012 | | Canada | | Qualitative, Case Study | | C | | • Capacity Building • Documenting and advancing PPI | | Flexible Approaches: Participatory planning was facilitated through focus groups and interviews. | | Member of advisory group | | • Improved research design • Multi-way Capacity Building  • Improved research design | |
| 78 | O'Hara | 2017 | | United Kingdom | | Mixed Methods | | I | | • Documenting and advancing PPI | | Participated in surveys, hackathon, expert panels, keynote speakers over a 3-day conference event. | | Member of working group | | • Improved research design | |
| 79 | Ostrow | 2017 | | United States | | Qualitative, Case Study | | C | | • Relevance of research  • Co-building | | Flexible Approaches: Participatory approaches were used throughout design, data collection analysis, and dissemination, included focus groups and surveys. | | Member of advisory group | | • Improved research design • Improved research design | |
| 80 | Portalupi | 2017 | | United States | | Qualitative | | I | | • Co-building | | Panel met for the first time to establish the group vision, advisors played a large part of determining the structure and direction of the panel. Meetings, every other month 1.5 -2-hour meetings with additional communication as needed. Key documents were created to support operations of the research advisory panel. | | Member of working group | | • Multi-way Communication and Collaboration • Multi-way Capacity Building | |
| 81 | Read | 2011 | | United Kingdom | | Qualitative, Case Study | | I | | • Documenting and advancing PPI | | Involvement of marginalized populations, extra attention and focus on the delivery and involvement of these individuals to meaningfully participate. Primarily focus groups and meeting. | | Member of steering committee | | • Improved research design | |
| 82 | Sauers-Ford | 2015 | | United States | | Mixed Methods | | I | | • Documenting and advancing PPI | | Parents made up the study team and were involved in the development of the project. Focus groups and phone interviews with many different families to inform different aspects of the research project. | | Member of steering committee | | • Improved research design | |
| 83 | Saunders | 2016 | | United States | | Mixed Methods | | I | | • Documenting and advancing PPI | | Involvement was supported by shared knowledge through research training, recognize and address power differentials by prioritizing relationships and trust and address logistics of stakeholder engagement through careful consideration of stakeholder meetings, communication and access capacity building sessions were held initially followed by 18 virtual meetings. | | Member of advisory group | | • Multi-way Communication and Collaboration • Inclusive Mechanisms and Processes | |
| 84 | Schenk | 2015 | | United States | | Qualitative | | I | | • Documenting and advancing PPI | | Involvement supported via multistakeholder focus groups | | Member of advisory group | | • Experiential knowledge valued as evidence • Multi-way Capacity Building | |
| 85 | Shippee | 2013 | | United States | | Review | | I | | • Documenting and advancing PPI | | Engagement was supported by meetings, emails, discussions and feedback via the panel. | | Member of advisory group | | • Improved research design | |
| 86 | Slade | 2010 | | United Kingdom | | Qualitative, Case Study | | I | | • Documenting and advancing PPI | | Involvement at start-up phase, primarily by meetings, emails, and discussions. | | Member of advisory group | | • Improved research design | |
| 87 | Snape | 2015 | | United Kingdom | | Mixed Methods | | C | | • Relevance of research  • Documenting and advancing PPI | | Participated in research activities, meetings, reviewing research materials including reports and papers for publication in peer reviewed journals and producing lay summaries. | | Member of advisory group | | • Multi-way Capacity Building  • Improved research design | |
| 88 | Tapp | 2017 | | United States | | Qualitative, Case Study | | C | | • Documenting and advancing PPI | | Various levels including: advising on study development; assisting with design and usability of study materials, including the toolkit, patient surveys and dissemination strategies; and advocacy via membership in external disease-specific organizations and participating in outcomes research conferences. | | Member of advisory group | | • Multi-way Capacity Building  • Multi-way Communication and Collaboration • Patient-informed and directed research • Experiential knowledge valued as evidence • A shared sense of purpose • Improved research design • Inclusive Mechanisms and Processes | |
| 89 | Taylor | 2018 | | United Kingdom | | Qualitative, Case Study | | C | | • Documenting and advancing PPI | | Group was established and met regularly for ten months. Ongoing engagement was promoted by the group taking responsibility for the rules, interactive and accessible activities, feeding back tangible impacts, ongoing contact, building a work ethic, joint celebrations, sessions with individual academic researchers. | | Member of research team | | • Multi-way Capacity Building  • Improved health outcomes  • A shared sense of purpose • Inclusive Mechanisms and Processes | |
| 90 | Vaillancourt | 2018 | | Canada | | Qualitative, Case Study | | I | | • Documenting and advancing PPI | | Surveys, patients as partners (provided feedback) and were involved in the decision-making process | | Member of advisory group | | • Improved research design | |
| 91 | Edwards | 2019 | | Canada | | Mixed Methods | | I | | • Co-building | | Email, teleconference, and full-day in-person meeting. | | Member of research team | | • Improved research design | |
| 92 | Wikman | 2018 | | Sweden | | Qualitative, Case Study | | C | | • Documenting and advancing PPI | | Participatory action research methodology was used supported by face-to-face workshops, meetings and related Web-based exercises. | | Member of advisory group | | • Improved research design • Multi-way Capacity Building  • Improved research design | |
| 93 | Williamson | 2015 | | United Kingdom | | Qualitative, Case Study | | C | | • Co-building | | Group meetings (nine in total), regular communication via email and information to support meetings. | | Member of advisory group | | • Improved research design • Multi-way Capacity Building | |
| 94 | Wilson | 2017 | | Canada | | Qualitative | | L | | • Co-building | | Conducted interviews, recruitment, and analyzed data | | Member of research team | | • NR | |
| 95 | Bryant | 2012 | | United Kingdom | | Qualitative | | L | | • Relevance of research  • Co-building | | Group discussions, reflective exercises and qualitative approaches were used to support a shared understanding. | | Member of research team | | • Patient-informed and directed research • Multi-way Communication and Collaboration • Multi-way Capacity Building  • Experiential knowledge valued as evidence | |
| 96 | Stevenson | 2019 | | United Kingdom | | Qualitative | | C | | • Relevance of research | | A 2-hour non-directive facilitated approach was used to make sense of the data as members of the research team. The scientific researchers followed the general guidance on communicating with people with dementia. They also conducted interviews with dementia patients which the co-researchers analyzed. Ideas were written up on a flipchart which served as a visual reminder for the group. | | Member of research team | | • Improved research design • Multi-way Capacity Building  • Experiential knowledge valued as evidence • Inclusive Mechanisms and Processes | |
| 97 | Mardsen | 2003 | | United Kingdom | | Qualitative | | C | | • Relevance of research | | Focus group discussions were organized with breast cancer patients to explore their attitude towards menopause. All discussions were tape recorded and transcribed to be analyzed further. There was also a half-day joint meeting for patients and clinicians to discuss any questions that they had for each other. this was done after the focus groups. Here they also talked about the key recommendations from the focus group discussions. Finally, before the national trial, two patients were asked to join a steering committee that decided how the national trial was going to be run. | | Member of advisory group | | • Inclusive Mechanisms and Processes • Multi-way Capacity Building  • Improved research design | |
| 98 | O'Brien | 2014 | | Australia | | Qualitative | | I | | • Relevance of research  • Documenting and advancing PPI • Capacity Building | | Advisory group, training, focus groups, meetings, team building, analyzed data over 4 years | | Member of advisory group | | • Multi-way Capacity Building  • Multi-way Communication and Collaboration • Experiential knowledge valued as evidence • Inclusive Mechanisms and Processes • A shared sense of purpose | |
| 99 | Healthtalk | 2014 | | United Kingdom | | News Release | | L | | • Documenting and advancing PPI | | NR | | NR | | • NR | |
| 100 | Afzal & Donnelly | 2014 | | United Kingdom | | Conference Poster | | I | | • Documenting and advancing PPI | | NR | | Member of advisory group | | • Improved research design • Multi-way Capacity Building  • Experiential knowledge valued as evidence | |
| 101 | Armitage & Crane | 2014 | | United Kingdom | | Conference Abstract | | C | | • Co-building • Relevance of research | | NR | | Member of steering committee | | • Experiential knowledge valued as evidence • Inclusive Mechanisms and Processes | |
| 102 | Bartram & Lawson | 2014 | | United Kingdom | | Conference Abstract | | I | | • Documenting and advancing PPI | | NR | | Member of advisory group | | • NR | |
| 103 | Bell | 2014 | | United Kingdom | | Conference Abstract | | L | | • Documenting and advancing PPI | | NR | | Member of advisory group | | • Improved research design • Patient-informed and directed research | |
| 104 | Beresford & Boote | 2014 | | United Kingdom | | Conference Abstract | | I | | • Documenting and advancing PPI | | NR | | Member of advisory group | | • NR | |
| 105 | Boaz | 2014 | | United Kingdom | | Conference Poster | | C | | • Documenting and advancing PPI | | NR | | NR | | • NR | |
| 106 | Boet | 2019 | | Canada | | News Release | | C | | • Impact on the research process | | NR | | NR | | • Inclusive Mechanisms and Processes • Experiential knowledge valued as evidence • Patient-informed and directed research | |
| 107 | Boulton | 2015 | | Canada | | Unpublished research report | | L | | • Relevance of research | | Patient researchers used a patient engagement research (PER) approach described by three phases: SET, COLLECT and REFLECT. Each phase was patient led and supported by focus groups. This circular approach to data collection and analysis serves to ensure meaningful patient involvement and contextual validity. | | Member of research team | | • Patient-informed and directed research • Multi-way Capacity Building  • Inclusive Mechanisms and Processes • Experiential knowledge valued as evidence | |
| 108 | Bourke & Knowles | 2014 | | United Kingdom | | Conference Abstract | | C | | • Relevance of research | | NR | | NR | | • NR | |
| 109 | Britt & Eglin | 2010 | | United Kingdom | | Conference Abstract | | I | | • Co-building | | NR | | Member of advisory group | | • NR | |
| 110 | Bryk | 2013 | | Canada | | Unpublished research report | | L | | • Relevance of research  • Capacity Building • Co-building | | Patient researchers used a patient engagement research (PER) approach described by three phases: SET, COLLECT and REFLECT. Each phase was patient led and supported by focus groups. This circular approach to data collection and analysis serves to ensure meaningful patient involvement and contextual validity. | | Member of research team | | • Patient-informed and directed research • Experiential knowledge valued as evidence • Multi-way Capacity Building  • Improved health outcomes | |
| 111 | Cameron | 2014 | | United Kingdom | | Conference Abstract | | C | | • Documenting and advancing PPI | | NR | | Member of working group | | • Inclusive Mechanisms and Processes | |
| 112 | Canadian Federation for Healthcare Improvement | 2017 | | Canada | | Unpublished research report | | C | | • Relevance of research | | Patient advisors are members of a steering committee and were invited to the roundtable to act as a resource and support for the patient advisors on the Better together advisory council | | Member of advisory group | | • Multi-way Capacity Building  • Experiential knowledge valued as evidence • Inclusive Mechanisms and Processes | |
| 113 | Choudhury & Wheeler | 2017 | | Canada | | Unpublished research report | | L | | • Co-building | | Patient researchers used a patient engagement research (PER) approach described by three phases: SET, COLLECT and REFLECT. Each phase was patient led and supported by focus groups. This circular approach to data collection and analysis serves to ensure meaningful patient involvement and contextual validity. | | Member of research team | | • Patient-informed and directed research • Experiential knowledge valued as evidence | |
| 114 | Dakers-Thomson | 2014 | | United Kingdom | | Conference Abstract | | I | | • Documenting and advancing PPI | | NR | | Member of advisory group | | • NR | |
| 115 | Culbert | 2018 | | Canada | | News Release | | C | | • Relevance of research | | NR | | Member of advisory group | | • Patient-informed and directed research | |
| 116 | Fairs | 2016 | | Canada | | Unpublished research report | | L | | • Relevance of research | | Patient researchers used a patient engagement research (PER) approach described by three phases: SET, COLLECT and REFLECT. Each phase was patient led and supported by focus groups. This circular approach to data collection and analysis serves to ensure meaningful patient involveent and contextual validity. | | Member of research team | | • Patient-informed and directed research • Experiential knowledge valued as evidence • Improved health outcomes  • Inclusive Mechanisms and Processes | |
| 117 | Mossie & Steel | 2014 | | United Kingdom | | Conference Abstract | | L | | • Documenting and advancing PPI | | NR | | NR | | • NR | |
| 118 | Gill | 2014 | | Canada | | Unpublished research report | | L | | • Relevance of research | | Patient researchers used a patient engagement research (PER) approach described by three phases: SET, COLLECT and REFLECT. Each phase was patient led and supported by focus groups. This circular approach to data collection and analysis serves to ensure meaningful patient involvement and contextual validity. | | Member of research team | | • Experiential knowledge valued as evidence | |
| 119 | Gill | 2017 | | Canada | | Unpublished research report | | L | | • Relevance of research | | Patient researchers used a patient engagement research (PER) approach described by three phases: SET, COLLECT and REFLECT. Each phase was patient led and supported by focus groups. This circular approach to data collection and analysis serves to ensure meaningful patient involvement and contextual validity. | | Member of research team | | • Patient-informed and directed research • Multi-way Capacity Building  • Inclusive Mechanisms and Processes • Experiential knowledge valued as evidence | |
| 120 | Gill | 2016 | | Canada | | Unpublished research report | | L | | • Relevance of research | | Patient researchers used a patient engagement research (PER) approach described by three phases: SET, COLLECT and REFLECT. Each phase was patient led and supported by focus groups. This circular approach to data collection and analysis serves to ensure meaningful patient involvement and contextual validity. | | Member of research team | | • Patient-informed and directed research • Multi-way Capacity Building | |
| 121 | Loud | 2012 | | United Kingdom | | Conference Abstract | | I | | • Documenting and advancing PPI • Impact on the research process  • Capacity Building | | One self-management intervention | | Member of advisory group | | • Multi-way Capacity Building  • Patient-informed and directed research | |
| 122 | Marlett | 2017 | | Canada | | Unpublished research report | | L | | • Relevance of research | | Patient researchers used a patient engagement research (PER) approach described by three phases: SET, COLLECT and REFLECT. Each phase was patient led and supported by focus groups. This circular approach to data collection and analysis serves to ensure meaningful patient involvement and contextual validity. | | Member of research team | | • Patient-informed and directed research • Multi-way Capacity Building | |
| 123 | Maybee | 2019 | | Canada | | Unpublished research report | | C | | • Relevance of research | | NR | | Member of research team | | • Patient-informed and directed research | |
| 124 | Miller & Teare | 2017 | | Canada | | Unpublished research report | | L | | • Co-building | | Patient researchers used a patient engagement research (PER) approach described by three phases: SET, COLLECT and REFLECT. Each phase was patient led and supported by focus groups. This circular approach to data collection and analysis serves to ensure meaningful patient involvement and contextual validity. | | Member of research team | | • Patient-informed and directed research • Experiential knowledge valued as evidence • Multi-way Capacity Building | |
| 125 | Miller & Teare | 2016 | | Canada | | Unpublished research report | | L | | • Co-building | | Patient researchers used a patient engagement research (PER) approach described by three phases: SET, COLLECT and REFLECT. Each phase was patient led and supported by focus groups. This circular approach to data collection and analysis serves to ensure meaningful patient involvement and contextual validity. | | Member of research team | | • Patient-informed and directed research • Multi-way Capacity Building  • Experiential knowledge valued as evidence • Multi-way Capacity Building  • Inclusive Mechanisms and Processes • Patient-informed and directed research | |
| 126 | Miller & Teare | 2015 | | Canada | | Unpublished research report | | L | | • Relevance of research | | Patient researchers used a patient engagement research (PER) approach described by three phases: SET, COLLECT and REFLECT. Each phase was patient led and supported by focus groups. This circular approach to data collection and analysis serves to ensure meaningful patient involvement and contextual validity. | | Member of research team | | • Patient-informed and directed research • Multi-way Capacity Building | |
| 127 | Miller | 2014 | | Canada | | Unpublished research report | | L | | • Relevance of research  • Co-building • Co-building | | Patient researchers used a patient engagement research (PER) approach described by three phases: SET, COLLECT and REFLECT. Each phase was patient led and supported by focus groups. This circular approach to data collection and analysis serves to ensure meaningful patient involvement and contextual validity. | | Member of research team | | • Improved health outcomes  • Experiential knowledge valued as evidence • Patient-informed and directed research • Multi-way Capacity Building | |
| 128 | Nunn | NR | | United Kingdom | | News Release | | L | | • Capacity Building | | Workshops | | NR | | • Multi-way Capacity Building | |
| 129 | Palm | 2012 | | United Kingdom | | Conference Abstract | | I | | • Co-building | | NR | | Member of advisory group | | • Multi-way Communication and Collaboration • Experiential knowledge valued as evidence | |
| 130 | Persaud & Shah | 2019 | | Canada | | News Release | | L | | • Impact on the research process | | NR | | Member of research team | | • NR | |
| 131 | Pritchard | 2012 | | United Kingdom | | Conference Poster | | C | | • Impact on the research process | | NR | | Member of working group | | • Multi-way Communication and Collaboration • Inclusive Mechanisms and Processes • Experiential knowledge valued as evidence | |
| 132 | Rainey & Morris | NR | | United Kingdom | | News Release | | L | | • Capacity Building • Co-building | | NR | | Member of advisory group | | • Multi-way Capacity Building  • Multi-way Communication and Collaboration • Patient-informed and directed research | |
| 133 | Roland | 2017 | | Canada | | Unpublished research report | | L | | • Relevance of research | | Patient researchers used a patient engagement research (PER) approach described by three phases: SET, COLLECT and REFLECT. Each phase was patient led and supported by focus groups. This circular approach to data collection and analysis serves to ensure meaningful patient involvement and contextual validity. | | Member of research team | | • Patient-informed and directed research • Inclusive Mechanisms and Processes • Experiential knowledge valued as evidence | |
| 134 | Shearkani | 2016 | | Canada | | Unpublished research report | | L | | • Relevance of research | | Patient researchers used a patient engagement research (PER) approach described by three phases: SET, COLLECT and REFLECT. Each phase was patient led and supported by focus groups. This circular approach to data collection and analysis serves to ensure meaningful patient involvement and contextual validity. | | Member of research team | | • Patient-informed and directed research • Multi-way Capacity Building  • Inclusive Mechanisms and Processes • Experiential knowledge valued as evidence | |
| 135 | Sheridan | 2014 | | Canada | | Unpublished research report | | L | | • Relevance of research | | Patient researchers used a patient engagement research (PER) approach described by three phases: SET, COLLECT and REFLECT. Each phase was patient led and supported by focus groups. This circular approach to data collection and analysis serves to ensure meaningful patient involvement and contextual validity. | | Member of research team | | • Patient-informed and directed research • Multi-way Capacity Building | |
| 136 | Taylor | NR | | United Kingdom | | News Release | | C | | • NR | | NR | | Member of advisory group | | • NR | |
| 137 | Aden | 2014 | | United Kingdom | | Conference Poster | | L | | • Documenting and advancing PPI | | NR | | NR | | • NR | |
| 138 | Johannesen | 2018 | | Canada | | Unpublished research report | | C | | • Relevance of research | | NR | | Member of advisory group | | • Experiential knowledge valued as evidence • Patient-informed and directed research • Inclusive Mechanisms and Processes | |
| 139 | CHSRF | 2012 | | Canada | | Video | | C | | • Impact on the research process | | NR | | Member of advisory group | | • NR | |
